# How Should We Measure? A Review of Circular Cities Indicators

**DOI:** 10.3390/ijerph19095177

**Published:** 2022-04-24

**Authors:** Andreea Loredana Bîrgovan, Elena Simina Lakatos, Andrea Szilagyi, Lucian Ionel Cioca, Roxana Lavinia Pacurariu, George Ciobanu, Elena Cristina Rada

**Affiliations:** 1 Institute for Research in Circular Economy and Environment “Ernest Lupan”, Calea Dorobantilor 71-73, 400609 Cluj-Napoca, Romania; loredana.birgovan@ircem.ro (A.L.B.); simina.lakatos@ircem.ro (E.S.L.); andrea.szilagyi@ircem.ro (A.S.); roxana.pacurariu@ircem.ro (R.L.P.); 2 Faculty of Industrial Engineering, Robotics and Production Management, Engineering and Management, Technical University of Cluj-Napoca, B-ul Muncii 103-105, 400609 Cluj-Napoca, Romania; 3 Faculty of Engineering, Lucian Blaga University of Sibiu, Blv. Victoriei 10, 550024 Sibiu, Romania; lucian.cioca@ulbsibiu.ro; 4 Academy of Romanian Scientists, 010071 Bucharest, Romania; 5 Faculty of Economics and Business Administration, University of Craiova, Str. Alexandru Ioan Cusa 13, 200585 Craiova, Romania; george.ciobanu@edu.ucv.ro; 6 Department of Theoretical and Applied Sciences, Insubria University of Varese, Via G.B. Vico 46, 21100 Varese, Italy

**Keywords:** circular economy, circular cities, circular indicators, smart urban metabolism

## Abstract

As the world continues to urbanize, it is necessary to identify and implement new urban development models and strategies in order to meet the challenges of sustainable development. As cities continue to face challenges in becoming fully circular, the need to establish a framework to measure the circular economy in urban areas grows. Many definitions for circular cities have been developed and addressed in recent years, as have numerous indicators. To make the transition to a circular city, we must integrate the findings and develop a general definition and measurement framework. This article aims at outlining a framework for circular cities indicators based on their key characteristics, as well providing directions for fostering circularity at the city level. To accomplish this goal, we conducted a systematic review and analyzed key papers published in the field of circular economy to determine how circular cities are measured. Choosing the right indicators to use for developing, monitoring, and evaluating circular cities is a difficult task for urban policymakers, managers, and planners. This highlights the significance of standardized frameworks for urban indicators. As a result, the authors propose a framework and highlight some key points about circular cities and smart urban metabolism.

## 1. Introduction

The majority of the world’s population currently lives in cities, with the 50% mark being broken in 2007. The global urban population share was 55% in 2018 [1], with the UN expecting a 68% share by 2050 [2]. Cities have a significant economic impact, accounting for over 70% of global GDP [3]. Cities consume more than 70% of all global resources energy produced, emit 70% of all greenhouse gases [4], and generate almost 70% of global waste [3].

The present linear model of production and consumption in modern society is unsustainable. It is urgent to address the socioeconomic issues of population increase and a growing middle class [5]. The circular economy (CE), which aims to design out waste, maximize resource value, reduce negative effects, and generate economic, environmental, and social capital [5,6], is gaining traction as a potential solution to these concerns.

Indicators to assess the efficiency of the circular economy model are necessary to support the transition towards the circular city model. Currently, there is not a set of indicators for assessing how a city is effective in moving towards circularity, nor are there tools for supporting it.

While acknowledging that many metrics have already been developed that could be used to measure circularity, the present review focuses on the frameworks, indicators, and indices specifically developed for the environment, economics, and social and smart technologies at a macro level.

## 2. The Importance of Circular Economy in Cities

### 2.1. Circular Economy and Sustainability

The concept of sustainable development presented by the World Commission on Environment and Development (WCED) in the report “Our Common Future”, often known as the Brundtland Report, is undoubtedly the most well-known definition: “sustainable development is growth that seeks to meet the needs of the present without compromising the ability of future generations to meet their own needs” [7]. This indicates that economic, social, and environmental sustainability are required.

In terms of circular economy, the researchers discovered 114 different definitions on 17 dimensions after analyzing the literature [8]. The Ellen MacArthur Foundation (EMF) provided the definition to which we are referring in this article. The EMF defines “an industrial economy that is restorative and renewable by intention and design” [9]. “The word “recovery” is key since the circular economy tries to heal earlier damage by developing better mechanisms inside the industry unit itself, not just as a preventative method to minimize pollution” [10]. This approach tries to activate efficient flows of materials, energy, labor, and information in order to restore natural and social capital, according to EMF’s definition, which combines both environmental and economic benefits through the notion of regenerative performance. McDonough and Braungart (in *Cradle to Cradle: Remaking the Way We Make Things*) divide materials into two categories: biological materials designed to safely re-enter the biosphere and contribute to the restoration of natural capital, and nonbiological materials [9,11].

The circular economy is an economic model that was created as an alternative to the linear economic model that is presently used in most industries. The linear model comprises the production and consumption of products and services without assigning significant weight to environmental externalities, such as waste generation and pollution resulting from the exploitation of virgin resources, a model in which economic goals take precedence. The circular economy, on the other hand, refers to the production and consumption of goods and services through closed-loop material flows that account for environmental externalities that occur during the design phase of products and goods with the goal of decoupling economic prosperity from resource consumption [12].

### 2.2. The Importance of Circular Cities

According to official data, the European Union generates more than 2.5 billion tons of waste each year. It is actively adjusting its waste management regulations in order to facilitate the transition to a more sustainable circular economy. In March 2020, the European Commission announced a new action plan for the circular economy, which includes ideas for a more sustainable product design, waste reduction, and consumer empowerment, as part of the European Green Agreement and in line with a planned new industrial strategy (repairs).

Parliament passed a resolution on the new circular economy action plan in February 2021, calling for additional measures to achieve a carbon-neutral, environmentally sustainable, nontoxic, and fully circular economy by 2050, including rigorous recycling rules and mandatory material use and consumption targets by 2030.

“You can’t improve what you can’t measure!” as the saying goes. There are four key objectives in measuring the state of the art, progress, and impact of a circular economy, according to the Organization for Economic Co-operation and Development (OECD) report “Circular Economy in Cities and Regions”, which looked at 51 cities and regions. The four key objectives are: raising awareness, supporting the circular economy, triggering actions, performance monitoring, and evaluation of results [13].

Over 90% of raw materials used around the world are not recycled, resulting in tremendous overexploitation of our planet’s finite natural resources and a harmful impact on our climate. Every year, Earth Overshoot Day, an annual measure of the planet’s overexploitation, gets closer. It fell on 29 July this year, although it was 23 September before the turn of the century. Greenhouse gas emissions and pollution have risen to historic levels, posing a threat to the planet’s overall well-being [14].

In their search for solutions, the forerunners of sustainability began to shift away from the widely held linear economy of products and services production and use (take, manufacture, discard) toward circular solutions (make, (re)use, recycle). By decreasing the usage of raw materials and keeping materials in the loop for as long as is feasible, circularity offers the potential to address our world’s global sustainability concerns. Every human-made product’s ecological footprint is reduced and minimized, protecting the world. According to UN Environmental Statistics, a fully circular economy could reduce resource use by 28% while also reducing carbon emissions by 72% [14].

Cities are growth engines that require monitoring and control. These are the primary causes of climate change, accounting for up to 76% of all carbon emissions. Despite occupying fewer than 2% of the Earth’s area, they account for 75% of world natural resource usage and 50% of global garbage generation. In terms of solutions, cities attract creative talent and so allow societal transformations toward sustainability in both the public and commercial sectors. Cities are among the most powerful entities that can impact development favorably if they become circular:

- The local government can encourage the reuse of building materials from its own construction, renovations, and demolition projects. As a result, the market for recycled building materials is stimulated.
- Grants, incentives, and tax exemptions can be used by local governments to encourage the development of new technology.
- Cooperation with other advanced cities to facilitate circular economy implementation. Supporting bottom–up efforts (idea competitions, startup funding).

An integrative definition for circular cities is the one synthesized by Paiho et al. (2020):

“A circular city is based on closing, slowing and narrowing the resource loops as far as possible after the potential for conservation, efficiency improvements, resource sharing, servitization and virtualization has been exhausted, with remaining needs for fresh material and energy being covered as far as possible based on local production using renewable natural resources”

[15]

Cities are one of the largest consumers of resources, but at the same time, they are incubators for innovation and have a huge potential to lead the transition to a circular economy. It has become really important to understand and define operable circular city indicators in order to understand where cities and citizens can have a real impact.

### 2.3. Circular Cities Indicators

The word “indicator” is derived from the Latin verb “indicare”, which means “to reveal” or “to indicate”, as well as “to announce” or “to make public”. According to Gallopin, indicators are valuable instruments for analyzing conditions and trends (even in connection to specific goals and targets), comparing places and situations, providing early warning information, and forecasting conditions and trends [16]. In terms of sustainability, indicators can help with communication by summarizing or simplifying key data, achieving perceptible phenomena of interest, and quantifying and measuring relevant data [16,17]. At various levels, such a communication role is expected to have a significant impact on supporting and improving political and decision-making processes [18].

In the context of circular cities, indicators can be used for monitoring, evaluation, and decision making. In reality, while establishing plans for the development and implementation of the circular economy, they can aid decision making by government officials and policymakers at both the local and national levels [19,20].

At the corporate level, however, indicators can assist managers in resolving operational difficulties, such as recognizing prospects for circular cities that are not currently (completely) explored and enhancing efficiency in utilizing existing synergies [21].

It is vital to build a measurement system in order to determine whether the CE principles result in meaningful changes [22]. “The ability to simplify, focus, and reduce the tremendous complexity of our dynamic environment into an easy to understand amount of useful information” is what indicators do [23]. Indicators, according to Church and Rogers [24], are “means of measuring change” that can be used to manage the transition to CE. Policy information, fostering literacy around the CE issue, allowing new quality standards, and comparing corporations for indices and sustainable investment markets could all benefit from CE indicators. In any case, Beratan et al. [17] emphasize the need of linking indicators to decision making and implementation. As a result, indicators are not sufficient in and of themselves to ensure a smooth transition to an CE, but they are a vital instrument in achieving this aim.

Many indicators are at the national level rather than at the level of the product [25,26]. The most high-profile of these comes from China, where the government uses well-known evaluation tools to analyze the effectiveness of its CE programs (e.g., life cycle assessment, ecoefficiency, and carbon footprint). Geng et al. (2013) [22] recognize that these indicators “weren’t designed for the closed-loop systemic feedback functions that characterize CE”.

The circular economy (CE) is being adopted by a number of countries throughout the world. Material flow analysis (MFA), energy analysis, and input–output analysis are currently used in macroscale CE monitoring [27]. In 2008, China became the first country to enact a particular law [28]; much of the CE literature pertains to China [29,30]. Germany and Japan have also been at the forefront of promoting the EC through real policy [22]. The European Union (EU) has agreed an action plan for the circular economy’s implementation towards the end of 2015 [31]. Other EU programs, such as resource efficiency [32] and waste legislation developed since the 1970s, previously included elements of the CE [33]. The European Commission (EC) recently suggested a circular economy monitoring framework [34]. The Ellen MacArthur Foundation (EMF) is credited for developing the concept of CE among private consultants [29].

Despite these efforts, there is no one-size-fits-all circular economy model at this time. Different players have different views of what CE can or should describe [35], and the connection to sustainability is not always obvious [8]. Despite the CE definition’s ambiguous constraints, particular procedures are required to track the CE’s progress. In this regard, indicators might be useful at various sizes of implementation and as a tool for assessing circularity [27,36].

However, because the concept is unclear and the indicators could lead to varied or even contradictory findings, what has to be monitored is being contested. Some authors have looked at the tools and approaches that have already been employed. Elia et al. [37] assessed a collection of procedures and indicators chosen from the European Environment Agency based on five features [38]. The authors pointed out that none of the indicators or approaches could track all of the characteristics. Iacovidou et al. [39] examined the strategies for evaluating waste resource recovery in order to enhance CE. Its findings revealed that none of the methodologies could explain the preservation of value in waste resources on their own, and that a comprehensive examination of the environmental, economic, social, and technical components of CE was required.

The monitoring framework for the circular economy is a concept for measuring development in the EU and its member states [34]. The indicators are divided into four categories under the “monitoring framework”: production and consumption, waste management, secondary raw materials, and competitiveness and innovation. Plastics, food waste, vital raw resources, building and demolition, and biomass and bio-based goods are all linked to the CE Action Plan for Europe’s priority areas [31]. There are ten indicators in the European Commission’s proposal, although six of them contain “sub-indicators”. The concept employs a total of twenty-four measuring guidelines. The metrics are based on data from Eurostat, the raw materials scoreboard, and the resource efficiency scoreboard, among other sources [34].

The indicators proposed by the European Commission are intended to assess and track the progress of the circular economy strategies announced by the European Union in the CE Action Plan 2015 [4], which measures four areas related to the various phases of the circular economy, including production and consumption, waste management, secondary raw resources, and competitiveness and innovation: all represented in the table below. As a result, the EC has released ten indicators, some of which are further subdivided into a set of sub-indicators, all of which are based on current Eurostat official statistics and backed up by additional official sources. Some metrics, such as green government procurement and food waste, are included despite the lack of a methodology.

## 3. Research Method

This study aims to see how the indicators of circular cities are distributed and to see which main themes are repeated frequently. This article aims at outlining a framework for circular cities indicators based on their key characteristics, as well as to further investigation and providing directions for fostering circularity at the city level. In order to do that, this study analyzes the most important research papers within the database of Web of Science (WOS). The goal is to see all the indicators and categories discussed in the research papers in order to understand the current situation in the academic world and how far the sector is from measuring circularity at the city level.

**Figure 1:**
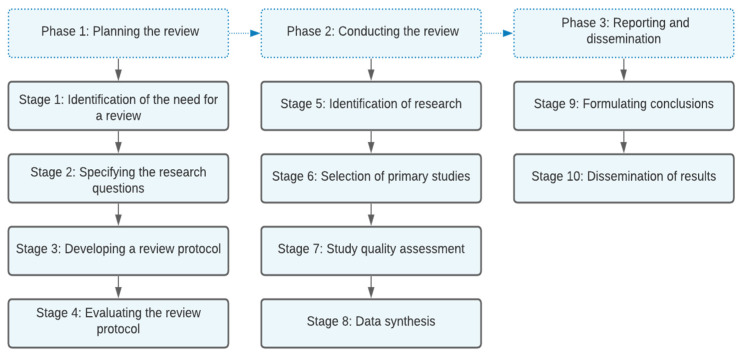
Systematic review steps adapted from Kitchenham [40].

We used the stages suggested by Kitchenham [40] and adapted them in order to perform a systematic review: (1) planning the review, (2) conducting the review, and (3) reporting and dissemination (Figure 1).

In order to provide an overview of how the scientific community responds to methodological issues in order to measure the circularity of cities, a review of the literature on the WOS search engine was conducted using keywords in different combination (circular economy, circular cities, circular indicators, smart cities indicators, etc.). The search focused on (1) scientific articles or book chapters published in English from 2010 to 2021; (2) topics of interest (circular economy indicators, circular cities, implementation of the circular economy at the city level, urban metabolism); (3) the comparison between the measurement of circular cities in Europe versus the world. Metrics available online without a published methodological background were not considered in the present study.

This report, which is structured in the form of a review of circular city indicators, consisted of an advanced search, with the aim of exploring the instruments used to measure circularity with a focus on the city. The search was followed by a screening process, performed by reading the title and summary of each result.

**Figure 2:**
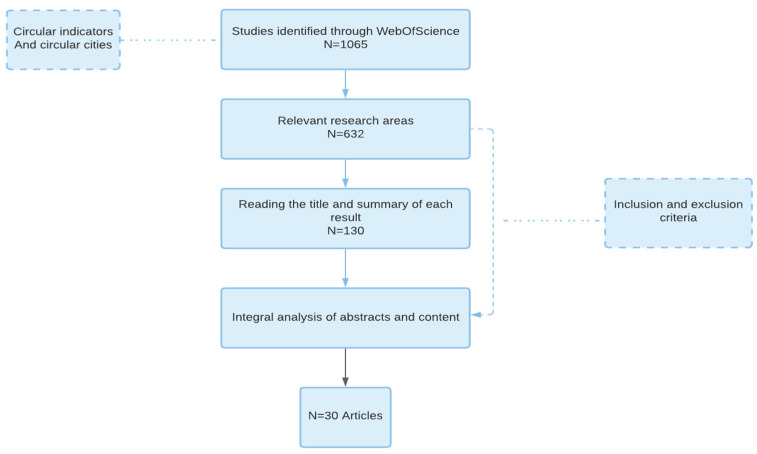
The study selection process.

The search returned 1065 results of which only 130 articles were considered the most representative at the first screening process (Figure 2). In the second step of the screening process, the authors read the abstract of every article which was selected in the first phase and decided to delete the following studies: (a) studies that did not directly address the issue of measuring circularity in a quantitative way or showed a high similarity to other articles already included in the review; (b) the articles which did not have developed or analyzed indicators at the city level; (c) the articles which were not presenting new ideas. After that, 30 articles remained. In addition to those articles, the authors decided to compare results with the action plan paper of European Union and add it to our review.

## 4. Results of the Review

Results came from examining research articles, including a series of other reviews in which indicators of circular cities were studied, both established by researchers in scientific journals and by analyzing a series of official documents and reports of cities that have adopted the circular city model. Thus, a number of interesting elements for aspects related to city measurement were observed after taking further steps in the transition to a circular economy.

Following our analysis, we discovered that there are various perspectives and definitions of circular cities. It would be extremely beneficial if everyone starts using a comprehensive definition that takes into account the various perspectives that researchers have when addressing circular cities. As a result of these various perspectives and definitions, we discovered that Paiho et al. [15] developed an integrative approach:

“A circular city is based on closing, slowing and narrowing the resource loops as far as possible after the potential for conservation, efficiency improvements, resource sharing, servitization and virtualization has been exhausted, with remaining needs for fresh material and energy being covered as far as possible based on local production using renewable natural resources”

[15]

However, even this definition which tried to synthetize more perspectives is referring mostly to the environmental dimension and it does not include the economic and social components. In order to make the definition more complex, we added to the above vision, as follows: It also integrates a way across all its functions in collaboration with researchers, citizens, and businesses in order to improve human well-being, reduce emissions, protect and enhance biodiversity, and promote social justice, in line with Sustainable Development Goals.

Additionally, not only were there many definitions for circular cities, this was also the case with indicators that were not very clearly defined or transparent. They often appear in articles, but there is no definition to understand exactly what they measure. To make the analysis easier, we attempted to categorize the information identified into four categories: environmental, social, economic, and smart.

Analyzing the indicators according to the framework proposed by us, it emerged that the indicators reported in scientific studies are primarily concerned with the environmental aspect, with little attention paid to the economic and social aspects. In the review conducted by Girard and Nocca [41], a total of 52 indicators assessing the environmental component (e.g., use of recycled goods in Municipal administration, amount of landfilled waste, recycling, rate of municipal waste, etc.) were identified, while only 6 indicators assessing the economic and financial components (spending on waste management, disposable income of households, reduction through reduced products and service costs, etc.) were identified (e.g., employment opportunities, job creation, active population in circular economy initiatives) with smart cities indicators.

In addition, we discovered some smart city indicators in the literature. These cities indicators, along with economic, environmental, and social indicators, are especially important at this time because we can now discuss smart urban metabolism. Considering the pandemic as well as the importance of technologies and digitalization, they should be incorporated into the measurement of circular cities.

Smart urban metabolism is a hybrid approach to developing smart and sustainable cities that takes into account technological, economic, environmental, and social perspectives. Smart urban metabolism becomes a strategic tool for decision makers, urban managers, and planners as a result of this feature. However, little is known about smart urban metabolism indicators.

To understand the functioning of cities and improve the efficiency of the processes that characterize urban metabolism, a holistic and multidimensional vision is required in order to support the following: waste management [42]; energy efficiency [43,44]; renewable energy sources [45,46]; water management [47]; cultural, social, and health aspects [48]; material flow [49,50]; biodiversity [51]; transport [52]; land use optimization [53]; air and noise pollution prevention [54]; environmental management systems [55]; and economic growth [56]—all within a resilient and technological context [57].

**Figure 3:**
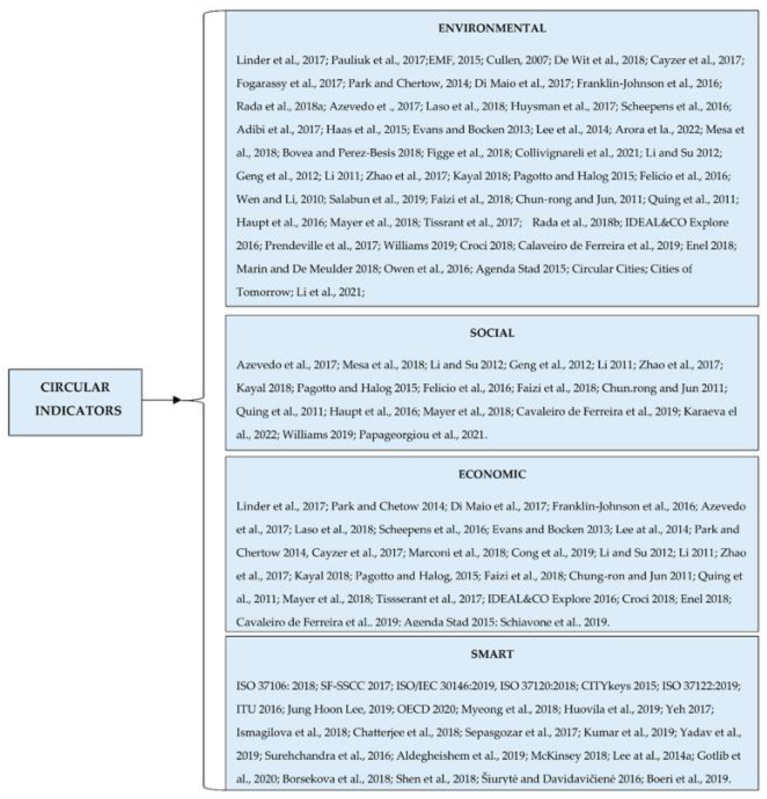
The reviewed studies on circular cities indicators [58,59,60,61,62,63,64,65,66,67,68,69,70,71,72,73,74,75,76,77,78,79,80,81,82,83,84,85,86,87,88,89,90,91,92,93,94,95,96,97,98,99,100,101,102,103,104,105,106,107,108,109,110,111,112,113,114,115,116,117,118,119,120,121,122,123,124,125,126,127,128,129,130,131,132,133,134,135,136].

Figure 3 was generated to organize all of the publications that established and addressed circular city indicators that measure the environmental, social, economic, and smart components [58,59,60,61,62,63,64,65,66,67,68,69,70,71,72,73,74,75,76,77,78,79,80,81,82,83,84,85,86,87,88,89,90,91,92,93,94,95,96,97,98,99,100,101,102,103,104,105,106,107,108,109,110,111,112,113,114,115,116,117,118,119,120,121,122,123,124,125,126,127,128,129,130,131,132,133,134,135,136].

However, the proportion of indicators identified in official documents and city-level reports is slightly different. As a result, a series of 96 environmental indicators were identified (e.g., recycling rate of municipal waste, use of recycled goods in municipal administration, public transportation usage, amount of reused resources, etc.), as well as 35 economic indicators (e.g., money saved in a year for an average household due to reducing the amount of products thrown away, budget allocated to stimulate pilot projects that employ circular economy at the local level; and environmental costs, such as cost of exhaustion, water pollution, CO_2_ emissions, toxicity, and land use); in addition, there were 46 social indicators identified (e.g., number of new green jobs, new business opportunities, number of training opportunities related to circular economy) and a series of standards and indicators for smart cities.

Circular cities can benefit from smart technologies in order to operate in a sustainable manner that meets the requirements of a circular economy. Intelligent electrical transportation, and green energy systems, for example, can be created using smart technologies to improve resource efficiency. Furthermore, smart technologies can aid in the creation of new jobs and the development of innovative products that can be easily recycled or repurposed without generating waste or releasing CO_2_ or toxins into the environment [137].

Many of the indicators identified in this study overlap, and we consider them more difficult to apply effectively in measuring a city and more difficult to manage by the authorities. Therefore, analyzing the indicators presented in Figure 3, we propose a series of indicators that appear most frequently in articles on the three dimensions: economic, environmental, and social. Our proposal is that the smart indicators should not be placed separately, considering that they address the three categories. We believe that the way we now talk about circular cities should overlap more with studies on intelligent urban metabolism. Thus, in Table 1, we can see our proposal of indicators. Of course, we selected these indicators, but we took into account another aspect, namely the seven pillars of the circular economy: materials are cycled at continuous high value; all energy is based on renewable sources; biodiversity is supported and enhanced through human activity; human society and culture are preserved; the health and well-being of humans and other species are structurally supported; human activities maximize the generation of societal value; and water resources are extracted and cycled sustainably.

## 5. Discussion

In order to facilitate the transition to a circular economy and to be able to make decisions in this direction, we need to understand the flow of materials and energy flows entering, being consumed, transformed, or stocked in and leaving cities, or, in other words, the urban metabolism. This means more specifically to use indicators to help us measure material, waste, water, and energy flows and stocks in cities and to have a clearer picture of the usefulness of infrastructure, stakeholders, and the elements that facilitate them. The fact that we can measure and have an overview of these loops can be a real help to see where we need to intervene and how we can connect the key elements. Additionally, in this way we can have a ranking system that can be used to measure the competitiveness of a city system and motivate cities to became circular.

Measuring cities’ performance in their transition to a circular economy allows cities to self-assess their achievements, identify barriers and opportunities, and adjust their development trajectory toward circularity accordingly. As a result of these considerations, the need for a solid and realistic framework of indicators for a circular economy transition in cities emerges. What is really missing, even though there are more and more studies in this direction, is a shared view on circular economy indicators among authorities and policy makers especially. There are data that every city can access more easily, so our initial proposal for a framework aims to have a core list of indicators that can fit every city.

For these reasons, we first tried through this study to provide a more complete definition and to define a simpler framework that can be applied in cities. However, of course, if we do not standardize such measuring instruments and do not have a series of similar assessments, it will be very difficult to compare cities and accelerate the transition.

This study has a number of limitations primarily due to the fact that it is a qualitative study, and even if we presented as the data transparently as possible, the way in which we selected the studies and the criteria used may include research bias. Additionally, only the articles in Web of Science have been included, and we may have omitted very relevant papers that do not appear in this database. Additionally, another limitation of this study is the fact that not all the indicators could be analyzed as accurately as possible because definitions were lacking in a large part of the articles. Another limitation is the combination of keywords we used in the way we built the database.

## 6. Conclusions

The circular economy (CE) is gaining attention, and monitoring frameworks and assessment tools are critical for documenting and measuring progress.

This systematic review examined the state of circular indicators at the city level, focusing on the issue of a large number of indicators that are currently more focused on the economic components in the scientific literature, with less emphasis on the social or environmental components and often exclude the smart technology factor.

Additionally, computerization, digitalization, and the efficiency of urban processes have become a priority for the development of residential areas, with the concept of “intelligent urban metabolism”/smart sustainable cities allowing us to achieve this goal [138,139]. In this sense, a city can be regarded as an open ecosystem characterized by a complex urban metabolism, owing to the numerous social, environmental, economic, governmental, and technological interactions between a wide range of internal and external actors [140,141,142,143].

This paper contributes to the theoretical development of the concept of circular cities by providing an integrated framework for circularity in cities and proposing some direction in order to make city measurement possible.

Circular cities indicators can be criticized for their lack of transparency and scientific basis. It was almost impossible to see how an indicator with similar label differs from another because of the lack of definitions. In order to solve this problem, we suggest that future studies standardize categories of indicators in order to have an appropriate level of transparency, quality, reliability, and consistency in their results. As a result, moving forward in measuring cities is extremely difficult if a widely accepted academic concept for the circular economy, circular cities, and indicators is not yet available. To figure out how to organize the indicators or how to use them to help with the transition to a more circular economy, we must first figure out what we want to monitor.

The authors believe that analyzing all of the criteria used to construct the indicators for circular cities would be beneficial to a future study as well. The authors tried to organize them by level of concern (environmental, economic, and social, using smart indicators at every level) in this review; however, in the literature we can see that there are many different ways in which these indicators have been organized and developed. We consider that the way we organize is simple and easy to use by everyone. Future studies could include more databases and articles to see which indicators are used the most according to the areas proposed by us and to improve this framework.

Future research should also consider using indicators that already exist and incorporating them into models such as Lakatos et al. [144], as well as the indicators for the 10R, to see how to achieve circular economy innovation at various levels.

Aside from the many different types of categories, we believe that having so many indicators can be a problem, making the implementation and measurement of cities more difficult and overwhelming.

## Figures and Tables

**Table 1:** Circular cities indicators.

Environment	Economic	Social
MFA Analysis	Budget allocated to stimulate pilot projects that employ circular economy at the local level	Livability/Quality of life ranking
Annual amount of CO_2_ emissions	Money granted to businesses or research projects linked to the circular economy	Employment opportunities, job creation. Number of new jobs (circular economy, green, recycling)
Amount or percentage of cycled material	Waste management costs	Number of training opportunities related to circular economy events
Amount of waste produced in the city	Environmental costs (costs of exhaustion, water pollution, CO_2_ emissions, toxicity, and land use in EUR per kilogram)	New business opportunities (that have integrated circularity into their development), number of local “green” companies
Use of renewable resource	E-government	Unemployment rate
Virgin resources used	Economic value of the resources used and the value at the time they are reintroduced into the system	Quality drinking water (population with access to safe drinking water)
Eco-car strategy—Municipal fleet powered by biogas, hydrogen, or electricity (including plug-in hybrids)	Green public procurement/e-procurement	Number of new circular initiatives
ICT infrastructure	Sales of locally produced goods	Environmental education (% of schools)
Smart buildings	Economic value of the resources used	Percent of population living below poverty line
Percentage of biodiversity	Resource usage: total raw material productivity	Percentage of inhabitants with housing deficiency in any of the following 5 areas: potable water, sanitation, overcrowding, deficient material quality, or lacking electricity
